# Standardization and performance evaluation of mononuclear cell cytokine secretion assays in a multicenter study

**DOI:** 10.1186/1471-2172-7-29

**Published:** 2006-12-12

**Authors:** Wayne G Shreffler, Cynthia M Visness, Melissa Burger, William W Cruikshank, Howard M Lederman, Maite de la Morena, Kristine Grindle, Agustin Calatroni, Hugh A Sampson, James E Gern

**Affiliations:** 1 Mount Sinai School of Medicine, Division of Pediatric Allergy & Immunology, New York, NY, USA; 2 Rho Inc., Chapel Hill, NC, USA; 3 Department of Pediatrics, University of Wisconsin Hospital, Madison, WI, USA; 4 Pulmonary Center, Boston University School of Medicine, Boston, MA, USA; 5 Department of Medicine, Johns Hopkins University School of Medicine, Baltimore, MD, USA; 6 Department of Pediatrics, Washington University School of Medicine, St. Louis, MO, USA

## Abstract

**Background:**

Cryopreservation of peripheral blood mononuclear cells has been used to preserve and standardize immunologic measurements for multicenter studies, however, effects of cryopreservation on cytokine responses are incompletely understood. In designing immunologic studies for a new multicenter birth cohort study of childhood asthma, we performed a series of experiments to determine the effects of two different methods of cryopreservation on the cytokine responses of cord and peripheral blood mononuclear cells.

**Results:**

Paired samples of PBMC were processed freshly, or after cryopreservation in a Nalgene container (NC) or a controlled-rate freezer (CRF). Although there were some differences between the methods, cryopreservation inhibited PHA-induced IL-10 secretion and *Der f *1-induced IL-2 secretion, and augmented PHA-induced IL-2 secretion and spontaneous secretion of TNF-α. In separate experiments, NC cryopreservation inhibited secretion of several cytokines (IL-13, IL-10, IFN-γ, TNF-α) by PHA-stimulated cord blood mononuclear cells. With the exception of PHA-induced IL-13, results from fresh and cryopreserved cord blood samples were not significantly correlated. Finally, in reproducibility studies involving processing of identical cell samples in up to 4 separate laboratories, variances in cytokine responses of fresh cells stimulated at separate sites did not exceed those in cryopreserved cells stimulated at a central site.

**Conclusion:**

Collectively, these studies indicate that cryopreservation can affect mononuclear cell cytokine response profiles, and that IL-10 secretion and antigen-induced responses may be especially vulnerable. These studies also demonstrate that mononuclear cell responses can be standardized for performance in a small number of laboratories for multicenter studies, and underscore the importance of measuring reproducibility and of testing whether cryopreservation techniques alter specific immunologic outcomes.

## Background

With the increase in multi-center studies has come an increased need to perform cellular studies that can be standardized. The responses of blood mononuclear cells are commonly used as surrogates for *in vivo *immune responses to adaptive and innate immune stimuli. Performing cellular assays at a single central laboratory by the use of cryopreserved and shipped specimens is generally thought to improve their feasibility and uniformity. This approach has been used extensively in vaccine and tumor immunology research, and several methodological investigations on the use of cryopreserved cells demonstrate preserved viability, as well as preserved functional responses such as proliferation and the frequency of cytokine producing cells – particularly to class I-restricted antigens [1-7]. In contrast, others have reported some loss of function in cryopreserved cells [8-10].

As part of the NIAID-sponsored Inner City Asthma Consortium, the Urban Environment & Childhood Asthma (URECA) investigation is a birth-cohort study examining the relationship between innate and adaptive immune responses, including responses to allergens, and the development of allergic asthma. There are four URECA clinical sites located in Baltimore, Boston, New York and St. Louis.

Few studies comparing the immunologic responses of fresh and cryopreserved mononuclear cells specifically examined responses to allergens and even fewer have assessed the impact of cryopreservation on the functional outcome of cytokine secretion [11]. Furthermore, as one of the primary benefits of centralization is thought to be decreased assay variability, we wished to directly compare assay variability using a 'peripheral lab' model in which each site obtains and stimulates freshly isolated mononuclear cells to that of a 'central lab' model, in which cells are locally cryopreserved and then shipped to a central site for stimulation assays.

We found that the response of cryopreserved peripheral blood mononuclear cells (PBMC) and cord blood mononuclear cells (CBMC) may not correlate with those of fresh cells. We also found that by careful standardization of laboratory procedures across participating laboratories, variability within the peripheral lab model is not different from that of the central lab model. These findings indicate the need to carefully evaluate effects of cryopreservation on immunologic outcomes and demonstrate that use of freshly isolated cells is a viable alternative for multicenter studies.

## Results

### Cytokine secretion of mitogen and antigen stimulated PBMC before and after cryopreservation

We first compared secretion of a panel of cytokines from cryopreserved and fresh atopic and non-atopic donor PBMC to innate (lipopolysaccharide [LPS]), polyclonal (phytohemaglutinin [PHA]) and adaptive (Der f 1, Bla g 2) immune cell stimuli in order to evaluate whether these responses are adequately preserved in cryopreserved cells. Accumulated IFN-γ, TNF-α, IL-10, IL-4, IL-5 and IL-2 were measured at several time points from supernatants of separate, parallel cultures (Additional File 1). LPS induced strong IL-10, TNF-α and IFN-γ responses that tended to peak on days 1 or 2. PHA strongly induced IFN-γ, TNF-α and IL-10, and lesser levels of IL-2 and IL-5. The major dust mite allergen, Der f 1, induced IL-2, TNF-α and IL-5 in some individuals. These responses were maximal on day 7. Responses to Bla g 2 were obscured by heavy endotoxin contamination and were not analyzed further.

**Figure 1:**
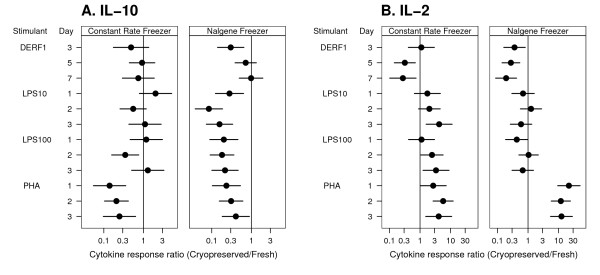
**Effect of cryopreservation on PBMC secretion of IL-10 and IL-2**. Mean response ratios were calculated for IL-10 (A) and IL-2 (B) for cells cryopreserved with either the Nalgene or constant rate freezer methods. Whiskers represent the 95% confidence intervals for difference. See methods for details of statistical analysis.

There were significant differences in cytokine secretion between fresh and cryopreserved cells for a number of stimulants (Additional File 1), and several responses appeared to be blunted. For example, PHA-induced IL-10 was approximately four to five-fold lower from cells cryopreserved using either of two methods. PHA-induced IL-10 peaked at day two with responses of 368 and 1,200 pg/ml (geometric means, p < 0.001) for Nalgene-preserved and fresh PBMC respectively. Cryopreservation in a constant rate freezer (CRF) produced similar effects. IL-10 responses to Der f 1 also were diminished by either method of cryopreservation (Figure 1A). LPS-induced IL-10 secretion was strongly blunted in Nalgene preserved PBMC, though this was not consistently seen in the CRF preserved cells.

In contrast, some responses were consistently increased in cryopreserved cells. Unstimulated PBMC cryopreserved by either method produced significantly greater (5–6 fold) amounts of TNF-α than fresh PBMC, although levels were low (<40 pg/ml) for all methods (Additional File 1). IL-2 secretion from PHA-stimulated cells was significantly greater in Nalgene PBMC (~10 fold), and the same effect (increase of 3–4 fold), was seen in the constant rate preserved cells (Figure 1B).

**Figure 2:**
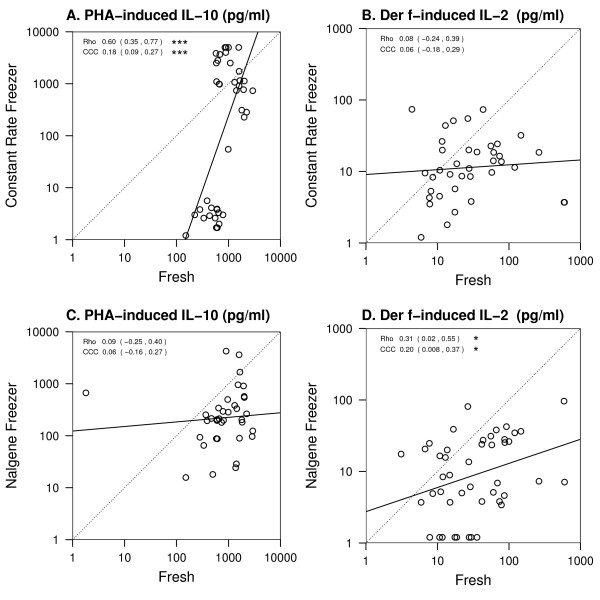
**Effect of cryopreservation on PBMC cytokine responses**. Data points represent cytokine responses by the same subjects' PBMC either cultured fresh or after cryopreservation. A, C. cells were stimulated for Each data point represents the comparison of the same subject at the same time point. Data shown include up to 3 time points per subject. The dotted line represents perfect agreement or concordance, and the solid line is best fit to data. Rho = Pearson correlation; CCC = Concordance Correlation Coefficient. See methods for details of statistical analysis. * p < 0.05; ** p < 0.01; ***p < 0.001.

Quantitative differences in cytokine secretion between fresh and cryopreserved cells may be acceptable if there is a predictable bias and qualitative differences are preserved. We therefore examined the correlation between stimulated fresh and cryopreserved cells. We assessed the quantitative differences between the different methods using two approaches: the Pearson correlation (rho) and the concordance correlation coefficient (CCC) [12]. The Pearson correlation measures a linear relationship, and the concordance correlation measures the absolute agreement between the two methods. As seen from representative scatter plots of secreted cytokine from fresh vs. cryopreserved cells from the same individuals stimulated with PHA (IL-10; Fig. 2A,C) or Der f 1 (IL-2; Figure 2B,D) there was generally poor concordance (CCC from 0.06 to 0.18 for PHA-induced IL-10 and 0.06 to 0.2 for Der f-induced IL-2, p < 0.05 only for CRF preserved PHA-induced IL-10) though for Nalgene preserved Der f-induced IL-2 and CRF preserved PHA-induced IL-10, there was statistically significant Pearson correlation (rho = 0.3 and 0.6, respectively). Poor correlation was also observed for other significantly affected responses, including PHA-induced IFN-γ, TNF-α, IL-5, IL-4 and IL-2 (Additional File 1).

Not all responses were altered as strongly. In particular, LPS-induced IFN-γ at both 10 and 100 ng doses were relatively well preserved with small differences only at some time points and good overall correlation (Additional File 1). However, the majority of measurable responses to PHA, LPS and allergens were significantly altered with poor correlation between fresh and cryopreserved cells.

### Effects of cryopreservation on cytokine secretion from LPS and PHA stimulated CBMC

**Figure 3:**
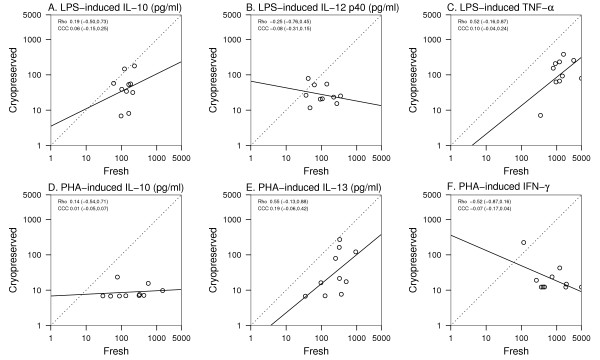
**Effect of cryopreservation on CBMC cytokine responses**. Data points represent cytokine responses by the same subjects' CBMC either cultured fresh or after cryopreservation. The dotted line represents perfect agreement or concordance, and the solid line is best fit to data. Rho = Pearson correlation; CCC = Concordance Correlation Coefficient. See methods for details of statistical analysis. * p < 0.05; **p < 0.01; ***p < 0.001.

We next compared the responses of fresh and cryopreserved cord blood mononuclear cells (CBMC). Freshly isolated CBMC secreted significant amounts of IFN-γ, TNF-α, IL-10 and IL-12 p40 in response to LPS (Additional File 2). Cryopreservation inhibited LPS-induced IL-10, IL-12 p40 and TNF-α (Figure 3A, B and 3C, respectively), and similar effects were noted for IFN-γ (data not shown). Cryopreservation also significantly inhibited PHA-induced IL-10, IL-13 and IFN = γ (Figure 3D, E and 3F).

Next, we analyzed the dataset to determine whether cryopreservation altered cytokine responses in a predictable fashion. In general, there was weak agreement between the fresh and cryopreserved cells with concordance correlations between -0.52 (-0.87 to 0.16; 95% confidence interval) for PHA-induced IFN-γ (Fig. 3F), and 0.19 (-0.06 to 0.42) for PHA-induced IL-13 (Fig. 3E). There were also no significant Pearson correlations between fresh and cryopreserved cell responses, although PHA-induced IL-13 from either fresh or preserved cells showed a trend toward linearity (rho = 0.55; -0.13 to 0.88, Fig. 3E).

**Figure 4:**
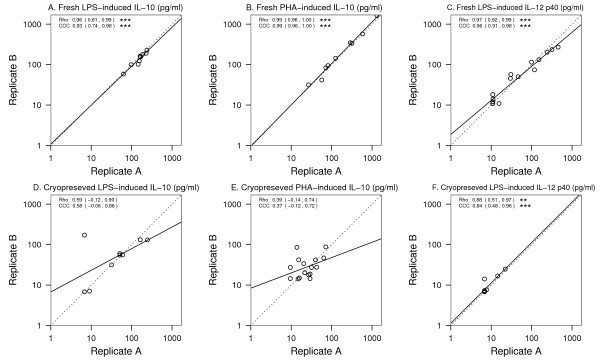
**Reproducibility of cytokine responses of fresh vs. cryopreserved cells**. Data points represent cytokine responses from replicate PBMC cultures. The dotted line represents perfect agreement or concordance, and the solid line is best fit to data. Rho = Pearson correlation; CCC = Concordance Correlation Coefficient. See methods for details of statistical analysis. * p < 0.05; ** p < 0.01; *** p < 0.001.

Finally, we examined the reproducibility of cytokine secretion when cells are stimulated fresh, or following cryopreservation (Figure 4). The reproducibility between replicate cultures of fresh cells for LPS-induced IL-10 (CCC = 0.93 [0.74 – 0.98], A), PHA-induced IL-10 (CCC = 0.99 [0.96 – 1.00], B) and LPS-induced IL-12 p40 (CCC = 0.96 [0.91 – 0.98], C) was strong. There was lower concordance, however, between replicate cultures of cryopreserved cells (CCC = 0.58 [-0.06 – 0.88], 0.37 [-0.12 – 0.72] and 0.84 [0.48 – 0.96] for LPS-induced IL-10, PHA-induced IL-10 and LPS-induced IL-12 p40, respectively, D-F).

### Performance of fresh vs cryopreserved mononuclear cells in a multicenter study

Although these data demonstrate that cryopreservation can affect PBMC cytokine secretion, cryopreservation and central laboratory cellular stimulation in the context of a multi-site study could minimize site-to-site variability, and this might outweigh disadvantages related to cryopreservation. In order to address this, we conducted a reproducibility study between the four URECA study sites and a central laboratory.

**Figure 5:**
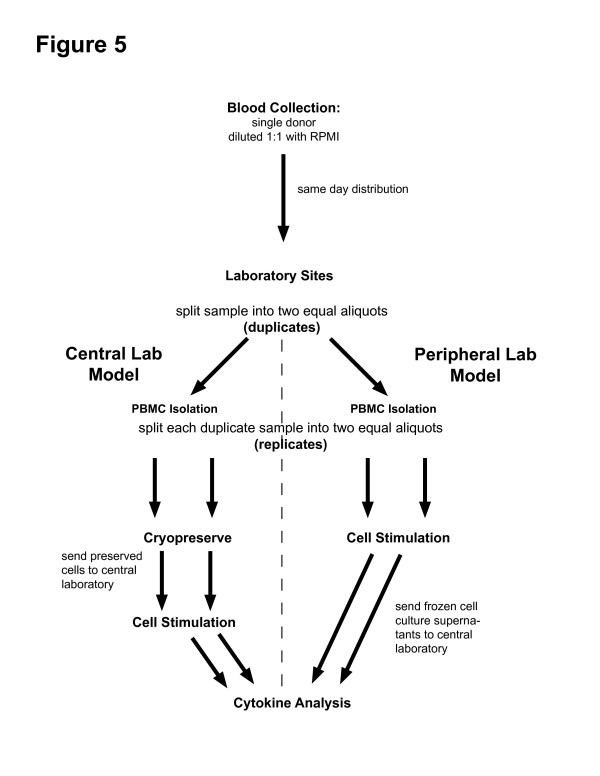
Four-site reproducibility study design.

On three separate occasions, blood from a single donor was collected, diluted 1:1 in RPMI, and transported to each site via commercial aircraft. Once received, half of the cells were stimulated (PHA, LPS, and tetanus toxoid [TT]) within 16 hours from the time of the blood draw (Figure 5). The other half were cryopreserved, shipped to the central laboratory, thawed and then stimulated in an identical fashion. Both the fresh and cryopreserved cells were processed in duplicate so that the variability of each protocol could be determined. This experimental design allowed us to directly address variability due to performance of stimulation assays at multiple sites versus the variability of the assay at a central site after cryopreservation.

**Figure 6:**
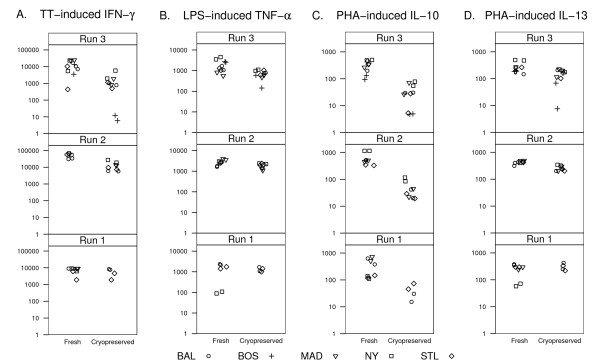
**Reproducibility of cytokine responses in central vs. peripheral processing protocols**. Cytokine responses from PHA, TT and LPS stimulated cells cultured at each local site as fresh cells or cultured centrally following cryopreservation and shipment from local sites. Units are pg/ml. Three separate experiments are shown as runs 1–3.

Cell recovery (mean 66%; range [49–77%]) and viability (mean 94.3%; [92.5–98%]) after cryopreservation were not significantly different between sites (not shown). Consistent with what was seen in the previous experiments, cytokine secretion was altered in cryopreserved cells (Table 1). For example, IL-10 responses were generally suppressed (Table 1 and Fig. 6C). PHA-induced IFN-γ and TNF-α secretion also were both significantly blunted (5 fold and 2 fold lower, respectively, Table 1), as was TT-induced IFN-γ (8-fold lower in preserved vs. fresh cells; Fig. 6A and Table 1).

**Table 1:** Comparison of PBMC cytokine secretion using central versus peripheral laboratory study models

	**IL-5^a^**	**IL-13^a^**	**TNF^a^**	**IFN-γ^a^**	**IL-10^a^**	**IL-8^b^**	**IL-12^a^**
**PHA**							
**- Peripheral lab (fresh)**	26 (8.7)	422* (1.9)	4,400* (3.0)	73,000* (1.4)	557* (0.9^#^)		
**- Central lab (cryopreserved)**	19 (8.0)	252* (3.3)	2,100* (2.0)	15,000* (4.0)	38* (7.1^#^)		

**LPS**							
**- Peripheral lab (fresh)**			2,600* (1.3)	68 (10)	981* (2.7)	332* (1.9)	7.9 (9.9^#^)
**- Central lab (cryopreserved)**			1,600* (5.2)	117(15)	708* (3.1)	141* (1.9)	6.6 (24^#^)

**TT**							
**- Peripheral lab (fresh)**	8.2 (6.0)	555 (5.3)	493* (13)	81,400* (8.8)	24* (3.1^#^)		
**- Central lab (cryopreserved)**	7.2 (26)	714 (2.5)	103* (1.4)	10,900* (1.6)	7* (20^#^)		

Values are geometric mean (^a^pg/ml, ^b^ng/ml); Percent coefficient of variation shown in parentheses. Red color and * indicates <0.01 for difference in cytokine produced between fresh v. cryopreserved PBMC; Blue color and # indicates <0.05 for difference in variance

Next, the variances of duplicate samples were compared for the two processing protocols. Only three comparisons of variances were found to be statistically different when comparing the peripheral and central laboratory methods: PHA-induced IL-10, LPS-induced IL-12, and TT-induced IL-10 (Table 1). For all three, there was greater variability in the centrally cultured cells that had been cryopreserved than in the fresh cells cultured at separate sites.

## Discussion

The Urban Environment and Childhood Asthma (URECA) study is investigating the relationship between early childhood exposures to allergens and viral infections and the development of asthma. Mononuclear cell cytokine responses to environmental antigens will be assessed over the first three years of life in a cohort of children at high risk for the development of asthma. The pilot studies reported here addressed the feasibility and optimal methodology for performance of those cellular assessments.

Assay standardization and inter-site variability are of great importance for multicenter studies. Several studies have documented substantial inter-site variation in assessing PBMC viability and responses following cryopreservation and shipment. For example, in a recent study comparing variability in cellular assays 20 × 10^6 ^cells were shipped from a central lab to 11 different sites. Cell recovery at those sites ranged from 4.7% to 114%. The viability ranged from 24.8% to 100% and coefficients of variation for HIV-1-specific IFN-γ secreting cells (spot forming cells per 10^6^) ranged from 36%–256% [13]. While the results from these laboratories were consistent with respect to internal ranking of responses (i.e. low, moderate, or high), a direct quantitative comparison between laboratories was not possible.

Since direct comparisons of quantitative measurements are essential for our multi-site study, we assessed multiple approaches to minimize variability. To quantify variability we measured cytokine production from cord blood and peripheral cells that were isolated and stimulated fresh with a panel of innate and adaptive stimulants, at each laboratory (peripheral lab model). These results were then compared to the results obtained using the same donor's cells that were isolated and preserved at each site and then shipped to a central lab where they were thawed and stimulated by an identical panel of stimulants (central lab model). Techniques for cell isolation, cell stimulation and cryopreservation, and all reagents were carefully standardized.

Using this approach, we determined that cytokine responses from cryopreserved mononuclear cells were significantly different from those of fresh cells. Cryopreservation reduced IL-10 secretion by PBMC in response to PHA, LPS or antigen (Der f 1) and also reduced IL-10 secretion from PHA- and LPS-stimulated CBMC. Suppression of PHA-induced IL-10 has been reported previously [14]. The strong and consistent suppression of IL-10 secretion from cryopreserved cells, even while other responses are preserved or enhanced, suggests that specific cellular subsets may be affected more strongly by cryopreservation. Some reports have suggested that antigen-presenting cells may be such a subset, [9,15,16] and several PBMC subsets with APC function may be potent producers of IL-10 including DCs, monocytes and B cells. Additionally, regulatory T cells may be an important source of IL-10 in these cultures and may have differential sensitivity to cryopreservation [17].

We next conducted a reproducibility study using cells from a single donor that were shipped to multiple sites. Protocols and reagents for the five sites had been standardized and all personnel had attended centralized training to minimize variability. Using this approach we demonstrated that with careful preparation and training, inter-lab variability was more similar in fresh cells processed in multiple laboratories than using centralized processing of cryopreserved cells in a single laboratory.

Many study designs seek to minimize variation by performing cellular assays at a single central laboratory using cells that are locally obtained, and shipped, usually after cryopreservation. This approach is valid so long as cryopreserved cell responses show a reasonable correlation with those of fresh cells. Several reports comparing fresh and cryopreserved PBMC responses have shown that some responses remain intact. Reimann et al. found that fewer than 10% of 27 PBMC samples lost proliferative responses to a panel of mitogens (PHA and PWM) and recall antigens (TT and *Candida*)^2^. Cryopreserved PBMC from renal carcinoma patients have been shown to retain proliferative capacity to IL-2, capacity to generate cytolytic activity, and secretion of PHA-induced IFN-γ^5^. Both of these studies were conducted at a single site and did not address the potentially deleterious impact of shipment and/or dry ice.

Some of the differences among experimental results obtained using cryopreserved cells are likely due to differences in techniques, protocols, and reagents that have been employed in different studies. For example, Disis et al. evaluated effects of storage on dry ice, specific media additives, and temperature at thawing on viability of cryopreserved PBMC [18]. In this study, cell viability was adversely affected by use of human AB serum in the media and by thawing of cells at 4°C rather than 25° or 37°C. For our study, several lots of AB serum were screened for effects on viability, and the viability of our cryopreserved samples was consistently above 90%. Nevertheless, we acknowledge that there may to be steps and/or reagents in our protocol that could be improved so that deleterious effects on PBMC cytokine responses could be lessened or even eliminated. The results of our own study, and previous studies, should be interpreted with the understanding that the protocols and technical details of both the cryopreservation protocol and the immunologic assays could influence comparability of results obtained using fresh vs. cryopreserved cells.

Similar to our study, several investigators have found blunted responses from cryopreserved cells, particularly to class II-restricted antigens. Weinberg et al.  found that cryopreservation blunted proliferative responses to some antigens TT and *Candida*, but not others (CMV and varicella-zoster virus) [19]. This may be due to a selective impact of cryopreservation on specific populations of antigen-presenting cells. This concept is supported by data from Maecker et al. who found that PBMC responses to pooled peptides were superior to whole antigen^9^.

Data are quite limited regarding the effects of cryopreservation on allergen-induced cytokine secretion. A small study of four patients found that IFN-γ, IL-2, IL-4 and IL-5 responses to a panel of antigens, including grass and dust allergens, were maintained in cryopreserved cells 11. However, these authors compared cytokine response by ELISPOT, and the analysis of allergen-specific responses was limited to a single subject.

## Conclusion

Based on our data, we have adopted the peripheral laboratory model for the URECA study. Our results indicate that with centralized training and standardized reagents and protocols, inter-laboratory variability of cytokine secretion assays at our four clinical sites could be reduced to levels comparable or better to that obtained using cryopreserved cells and central processing. This approach also eliminates alterations of cytokine response profiles created by our method of cryopreservation. Collectively, these findings underscore the need to carefully assess the effects of cryopreservation, processing protocols and shipment of cellular specimens on specific outcomes of interest.

## Methods

### Standardization procedures

All reagents were purchased in bulk. After dose response experiments of the stimuli were completed, single-use aliquots for the entire study were frozen (-80°C) and shipped to each site laboratory on dry ice. Uniform sources were established for plasticware and other consumable reagents, and sites used the same model centrifuge (Allegra 50, Beckman). Quality control procedures were established for calibration and maintenance of incubators (CO_2 _and temperature) centrifuges, pipettes, refrigerators and freezers.

Standard procedures were developed for cell isolation and stimulation for cytokine production. Two training sessions were held: head technicians from each of the clinical sites reviewed and practiced the cell separation and cryopreservation procedures in February 2004, and in May 2005 lead technicians and immunologists practiced the cell separation, cryopreservation and cell stimulation procedures. Performance criteria were developed for cell yield and viability, and all technicians were certified in these techniques.

### Study subjects

The study subjects who donated blood for pilot studies (Figures 1, 2; Additional File 1) were healthy volunteers. CBMC samples were from the first 10 subjects recruited to the URECA study (Figures 3, 4). Each of these children had at least one allergic parent, lived in urban neighborhoods selected for low socioeconomic status, and were free from congenital anomalies or respiratory distress at birth. The reproducibility experiments involving each of the study center laboratories were carried out using PBMC from a single non-allergic healthy normal volunteer (Figure 6; Table 1). All study subjects or their surrogates gave written informed consent and the ethical review boards at each institution approved all studies.

### Isolation and cryopreservation of mononuclear cells

Mononuclear cells were obtained from peripheral or cord blood by density gradient separation. Whole blood was diluted 1:1 with RPMI containing heparin at the time of collection and this was overlaid on Ficoll Paque Plus (Amersham Biosciences) using Accuspin tubes (Sigma). Cells were washed with PBS containing 1% human AB serum.

The cryopreservation procedure was adapted from a protocol developed by the Immune Tolerance Network, and was kindly provided by Dr. Jeff Bluestone. Washed mononuclear cells were gently resuspended in human AB serum at room temperature to a concentration of 2 × 10^7^/mL. The serum used in these experiments was prescreened for its capacity to preserve cell viability and PHA-induced IFN-γ responses in preliminary experiments. An equal volume of 20% DMSO in human AB serum was slowly added (over approximately two minutes) to bring cells to 1 × 10^7^/mL. These were then transferred to isopropanol-containing freezing containers (Nalgene) at room temperature and placed in a -80°C freezer for storage pending transfer to dry ice for shipment to the central laboratory. During pilot studies we also compared the use of a constant rate freezer (Thermo) programmed to -1°C/hour from room temperature to -80°C, after which cells were shipped as above on dry ice.

### Cell stimulation assays

Following isolation from whole blood, fresh mononuclear cells were resuspended in AIM-V media (Invitrogen Corp., Carlsbad, CA) at a concentration of 4 × 10^6 ^cells/ml. Cryopreserved mononuclear cells were quick-thawed in a 37°C water bath until a small piece of ice remained and then transferred to a 15 ml tube. Cold RPMI media was slowly added drop-wise, mixing with each addition, to a volume of 10 ml. Cells were centrifuged at 300 × *g *for 10 minutes, washed with 10 ml RPMI, counted by dye exclusion with trypan blue for viability and resuspended in AIM-V media at a concentration of 4 × 10^6 ^cells/ml.

To 5 ml culture tubes, 250 μl (1 × 10^6 ^cells) of either fresh or thawed mononuclear cell suspension was added. Stimulants were then added to each tube (250 μl) at the following final concentrations: 15 μg/ml PHA; 100 or 10 ng/ml LPS (including 5% human AB serum); 10 μg/ml Der f 1, 10 μg/ml Bla g 2, 10 μg/ml tetanus toxoid (TT). AIM-V medium (250 μl) was added to control cultures. Sufficient quantities of stimulants were prepared from single lots at the central laboratory, shipped to each laboratory, and stored at -80°C until used. Based on kinetics of responses shown in the pilot PBMC studies, CBMC and reproducibility experiments were cultured for 1 day (PHA, LPS, and media control) or 5 days (TT and media control) at 37°C in a 5% CO2 humidified incubator. Tubes were centrifuged at 400 × *g *for 10 minutes, and supernatants were aliquoted and stored at -80°C.

### Four site reproducibility studies

To monitor the performance of procedures in the URECA laboratories, a four-site reproducibility study was performed on 3 separate occasions (Figure 5). For this study, a single aliquot of blood (~160 mL) was drawn from a volunteer, and was mixed with an equal volume of RPMI. This mixture was divided into 40 mL aliquots, and two aliquots each were packed in Styrofoam boxes along with "cold packs" warmed to room temperature. The packages were sent to each of the four clinical laboratories via same-day air courier service in temperature-controlled compartments, or for the second session, the packages were carried in the passenger compartment by technicians. Processing of the samples began simultaneously once after all of the packages had arrived at these destinations.

PBMCs were isolated from each of the two aliquots. Cells from the first aliquot were divided into two replicates, and stimulated for measurement of cytokine secretion. The culture supernatants were frozen (-80°C) and then shipped to Madison for cytokine analysis. Cells from the second aliquot were divided into two replicates which were cryopreserved and held at -80°C pending shipment to Madison on dry ice. These replicates were thawed within 2 weeks of receipt, and the cells were then stimulated as described above.

### Measurement of cytokines

Culture supernatants from fresh and frozen mononuclear cells were assayed for various cytokines using the Beadlyte^® ^Human Multi-Cytokine Flex Kit (Upstate, Lake Placid, NY), a bead-based sandwich immunoassay. Protocol A of the manufacturer's instructions were followed, and cytokines were quantified using the Luminex^® ^100™ instrument and IS 2.3 software (Luminex Corporation, Austin, TX). Pilot study samples were obtained using BD cytokine bead array (CBA) reagents (BD Biosciences, San Jose, CA) according to manufacturer's protocol, acquired on a BD LSR II and analyzed using BD CBA software.

### Statistics and Analysis

For Figure 1, each cytokine was analyzed using a linear mixed model [20] with individuals as random effects and condition, stimulant, and day as fixed effects. All cytokine measures were log-transformed to better meet the normal distribution outcome assumption of the linear mixed model. Heterogeneous variances for each condition were determined using the likelihood ratio test. Estimation was based on the method of restricted maximum likelihood (REML). The mean difference, and its 95% confidence interval, between cryopreserved (both constant rate freezer and Nalgene freezer) and fresh PBMC cytokine secretion was calculated for each combination of condition, stimulant, and day.

We assessed the quantitative differences between fresh vs. cryopreserved samples from the same patient or from replicates using two approaches: the Pearson correlation and the concordance correlation ^12^. The Pearson correlation measures a linear relationship, and the concordance correlation measures the absolute level of agreement between two methods in relation to the 45° identity line.

All analyses were performed using SAS software (SAS Institute, Inc. Cary, NC). Figures were produced in R [21].

## Authors' contributions

WGS, WWC and JEG drafted the manuscript with input and edits from all authors. Experiments and analysis were planned and carried out by WGS, MB, WWC, HML, KG, MM, HAS, JEG. CMV and AC provided additional statistical analyses and produced the figures.

## Supplementary Material

Additional file 1 Supplemental Table 1 – Cytokine from fresh and cryopreserved PBMC pilot study. Complete data set of secreted cytokine from fresh and cryopreserved PBMC culture. INS = insufficient number of paired samples for this analysis; ND = majority of cytokine levels below detection; rho = pearson correlation; CCC = concordance correlation. See methods for details of statistical analyses. Click here for file

Additional file 2 Supplemental Table 2 – Cytokine from fresh and cryopreserved CBMC. Complete data set of secreted cytokine from fresh and cryopreserved CBMC culture. N = number of samples; (below limit) = number below detectable level for indicated stimulant/cytokine; SD = standard deviation; Rho = Pearson correlation; CCC = Concordance Correlation Coefficient. See methods for details of statistical analysis. Click here for file

## References

[B1] Disis ML, Maecker HT, Clay TM, Lyerly HK, Chang JC, Lotze MT, Thompson AW (2003). Immunologic monitoring to assess immunity to solid tumors. Measuring Immunity: the immunologic surrogates handbook.

[B2] Reimann KA, Chernoff M, Wilkening CL, Nickerson CE, Landay AL (2000). Preservation of lymphocyte immunophenotype and proliferative responses in cryopreserved peripheral blood mononuclear cells from human immunodeficiency virus type 1-infected donors: implications for multicenter clinical trials. The ACTG Immunology Advanced Technology Laboratories. Clin Diagn Lab Immunol.

[B3] Maecker HT, Moon J, Bhatia S, Ghanekar SA, Maino VC, Payne JK, Kuus-Reichel K, Chang JC, Summers A, Clay TM, Morse MA, Lyerly HK, DeLaRosa C, Ankerst DP, Disis ML (2005). Impact of cryopreservation on tetramer, cytokine flow cytometry, and ELISPOT. BMC Immunol.

[B4] Kleeberger CA, Lyles RH, Margolick JB, Rinaldo CR, Phair JP, Giorgi JV (1999). Viability and recovery of peripheral blood mononuclear cells cryopreserved for up to 12 years in a multicenter study. Clin Diagn Lab Immunol.

[B5] Sobota V, Bubenik J, Indrova M, Vlk V, Jakoubkova J (1997). Use of cryopreserved lymphocytes for assessment of the immunological effects of interferon therapy in renal cell carcinoma patients. J Immunol Methods.

[B6] Weinberg A, Zhang L, Brown D, Erice A, Polsky B, Hirsch MS, Owens S, Lamb K (2000). Viability and functional activity of cryopreserved mononuclear cells. Clin Diagn Lab Immunol.

[B7] Oldham RK, Dean JH, Cannon GB, Ortaldo JR, Dunston G, Applebaum F, McCoy JL, Djeu J, Herberman RB (1976). Cryopreservation of human lymphocyte function as measured by in vitro assays. Int J Cancer.

[B8] Costantini A, Mancini S, Giuliodoro S, Butini L, Regnery CM, Silvestri G, Montroni M (2003). Effects of cryopreservation on lymphocyte immunophenotype and function. J Immunol Methods.

[B9] Maecker HT, Dunn HS, Suni MA, Khatamzas E, Pitcher CJ, Bunde T, Persaud N, Trigona W, Fu TM, Sinclair E, Bredt BM, McCune JM, Maino VC, Kern F, Picker LJ (2001). Use of overlapping peptide mixtures as antigens for cytokine flow cytometry. J Immunol Methods.

[B10] Jennes W, Kestens L, Nixon DF, Shacklett BL (2002). Enhanced ELISPOT detection of antigen-specific T cell responses from cryopreserved specimens with addition of both IL-7 and IL-15 – the Amplispot assay. J Immunol Methods.

[B11] Kreher CR, Dittrich MT, Guerkov R, Boehm BO, Tary-Lehmann M (2003). CD4+ and CD8+ cells in cryopreserved human PBMC maintain full functionality in cytokine ELISPOT assays. J Immunol Methods.

[B12] Lin L, I-K (1989). A concordance correlation coefficient to evaluate reproducibility. Biometrics.

[B13] Cox JH, Ferrari G, Kalams SA, Lopaczynski W, Oden N, D'Souza MP (2005). Results of an ELISPOT proficiency panel conducted in 11 laboratories participating in international human immunodeficiency virus type 1 vaccine trials. AIDS Res Hum Retroviruses.

[B14] Venkataraman M (1996). Effects of cryopreservation on immune responses: IX. Stimulus-mediated dichotomy in IL-10 production by frozen human peripheral blood mononuclear cells. J Hematother.

[B15] Makino M, Baba M (1997). A cryopreservation method of human peripheral blood mononuclear cells for efficient production of dendritic cells. Scand J Immunol.

[B16] Stevenson HC, Bonvini E, Favilla T, Miller P, Akiyama Y, Hoffman T, Oldham R, Kanapa D (1984). Characterization of purified cryopreserved human monocyte function in assays of superoxide production, accessory cell function, chemotaxis, and in fluorescent cell sorter analysis. J Leukoc Biol.

[B17] Milson TJ, Keller RH (1982). The variable effect of cryopreservation on peripheral blood mononuclear populations. J Clin Lab Immunol.

[B18] Disis ML, dela Rosa C, Goodell V, Kuan LY, Chang JC, Kuus-Reichel K, Clay TM, Kim Lyerly H, Bhatia S, Ghanekar SA, Maino VC, Maeker HT (2006). Maximizing the retention of antigen specific lymphocyte function after cryopreservation. J Immunol Methods.

[B19] Weinberg A, Betensky RA, Zhang L, Ray G (1998). Effect of shipment, storage, anticoagulant, and cell separation on lymphocyte proliferation assays for human immunodeficiency virus-infected patients. Clin Diagn Lab Immunol.

[B20] Verbeke G, Molenberghs G (2001). Linear Mixed Models for Longitudinal Data.

[B21] R_Development_Core_Team (2005). R: A language and environment for statistical computing.

