# Supplementary material for: Standardization and performance evaluation of mononuclear cell cytokine secretion assays in a multicenter study
Source: BMC Immunol. 2006 Dec 12;7:29. doi: 10.1186/1471-2172-7-29 (PMC1762025; doi:10.1186/1471-2172-7-29)
Supplement: Additional file 1 — Supplemental Table 1 – Cytokine from fresh and cryopreserved PBMC pilot study. Complete data set of secreted cytokine from fresh and cryopreserved PBMC culture. INS = insufficient number of paired samples for this analysis; ND = majority of cytokine levels below detection; rho = pearson correlation; CCC = concordance correlation. See methods for details of statistical analyses. [file 1471-2172-7-29-S1.pdf]

Supplemental Table 1. - Cytokine from fresh and cryopreserved PBMC.

Stimulant	cytokine	Day	N Fresh	Geometric mean-fresh	N CRF	Geometric mean-CRF	p-value for the ratio of geometric means Fresh/ CRF	correlation Fresh/ CRF	N Nalgene	Geometric mean-Nalgene	p-value for the ratio of geometric means Fresh/ CRF	correlation Fresh/ Nalgene
MEDIUM	IFN	1	16	**7.4**	10	**16.3**	0.304		10	**2.1**	0.072	
		2	20	**6.5**	20	**12.4**	0.288		15	**3.3**	0.243	
		3	16	**11.3**	10	**33.7**	0.153	rho=0.18(-0.08,0.42)	11	**3.8**	0.11	rho=0.08(-0.18,0.33)
		5	21	**10.3**	15	**12.9**	0.721	CCC=0.17(-0.08,0.40)	20	**11.7**	0.811	CCC=0.08(-0.17,0.32)
		7	5	**5.7**	10	**12.0**	0.487		9	**6.8**	0.87	
	TNF	1	16	**12.8**	10	**18.9**	0.484		10	**22.0**	0.317	
		2	20	**4.6**	21	**28.0**	<0.001		15	**22.0**	<0.001	
		3	16	**4.4**	10	**33.1**	<0.001	rho=0.13(-0.12,0.37)	11	**8.2**	0.235	rho=0.37(0.13,0.57)
		5	21	**3.6**	16	**16.8**	<0.001	CCC=0.09(-0.08,0.25)	20	**18.0**	<0.001	CCC=0.21(0.06,0.34)
		7	5	**4.3**	10	**36.7**	0.008		9	**10.0**	0.297	
	IL-10	1	16	**9.9**	10	**6.8**	0.438		10	**2.5**	0.001	
		2	20	**5.1**	21	**7.8**	0.233		15	**3.0**	0.13	
		3	16	**4.2**	9	**4.4**	0.904	ND	11	**2.6**	0.251	ND
		5	21	**4.5**	16	**6.4**	0.341		20	**3.5**	0.422	
		7	5	**8.2**	11	**7.3**	0.833		9	**5.7**	0.498	
	IL-5	1	16	**1.9**	10	**2.7**	0.43		10	**1.8**	0.815	
		2	20	**1.7**	21	**2.4**	0.299		15	**2.0**	0.685	
		3	16	**2.3**	10	**2.8**	0.621	ND	11	**2.0**	0.777	ND
		5	21	**2.3**	16	**3.9**	0.146		20	**2.7**	0.552	
		7	5	**2.6**	11	**4.6**	0.304		9	**3.8**	0.475	
	IL-4	1	16	**2.3**	10	**2.5**	0.757		10	**1.9**	0.525	
		2	20	**2.5**	21	**1.6**	0.077		15	**2.3**	0.734	
		3	16	**2.7**	10	**2.7**	0.997	ND	11	**2.7**	0.961	ND
		5	21	**2.6**	16	**1.8**	0.166		20	**2.3**	0.514	
		7	5	**1.5**	11	**1.3**	0.678		9	**2.4**	0.241	
	IL-2	1	16	**5.2**	10	**7.9**	0.399		10	**3.3**	0.288	
		2	20	**6.4**	21	**6.2**	0.947		15	**4.5**	0.349	
		3	16	**11.4**	10	**12.8**	0.812	ND	11	**4.3**	0.02	ND
		5	21	**12.6**	16	**7.8**	0.246		20	**6.4**	0.047	
		7	5	**8.7**	11	**6.4**	0.644		9	**5.8**	0.518	
PHA	IFN	1	11	**253.7**	5	**15.8**	0.007		4	**3132.0**	0.01	
		2	12	**3051.4**	14	**2757.0**	0.894	rho=0.67(0.02,0.92)	6	**12690.2**	0.092	rho=0.43(-0.23,0.82)
		3	10	**2292.3**	5	**31.9**	<0.001	CCC=0.40(0.03,0.67)	5	**19433.9**	0.022	CCC=0.35(-0.16,0.72)
	TNF	1	8	**3874.7**	5	**26.4**	<0.001		4	**3009.9**	0.758	
		2	13	**2556.4**	14	**483.4**	0.003	rho=0.61(0.01,0.88)	8	**2919.9**	0.826	rho=-0.57(-0.85,-0.06)
		3	12	**829.1**	5	**10.9**	<0.001	CCC=0.12(-0.02,0.25)	7	**5159.9**	0.004	CCC=-0.53(-0.81,-0.06)
	IL-10	1	16	**584.2**	10	**81.8**	<0.001		10	**136.5**	<0.001	
		2	20	**1200.5**	21	**249.9**	<0.001	rho=0.59(0.34,0.76)	15	**368.6**	<0.001	rho=0.10(-0.24,0.41)
		3	16	**582.1**	10	**144.4**	0.004	CCC=0.19(0.09,0.28)	11	**235.3**	0.024	CCC=0.06(-0.15,0.27)
	IL-5	1	16	**8.7**	10	**11.0**	0.604		10	**35.9**	<0.001	
		2	20	**37.0**	21	**111.7**	<0.001	rho=0.14(-0.18,0.43)	15	**178.1**	<0.001	rho=0.31(-0.02,0.58)
		3	16	**35.1**	10	**45.7**	0.553	CCC=0.11(-0.15,0.36)	11	**161.0**	<0.001	CCC=0.24(-0.02,0.47)
	IL-4	1	16	**13.9**	10	**13.0**	0.85		10	**28.6**	0.005	
		2	20	**26.1**	21	**37.4**	0.163	rho=-0.55(-0.73,-0.29)	15	**33.1**	0.29	rho=0.10(-0.23,0.42)
		3	16	**12.3**	10	**16.9**	0.353	CCC=-0.52(-0.71,-0.27)	11	**22.8**	0.015	CCC=0.09(-0.21,0.38)
	IL-2	1	16	**53.5**	10	**144.3**	0.048		10	**1168.6**	<0.001	
		2	20	**79.8**	21	**454.4**	<0.001	rho=-0.69(-0.83,-0.49)	15	**949.2**	<0.001	rho=0.20(-0.14,0.50)
		3	16	**59.2**	10	**241.2**	0.005	CCC=-0.55(-0.73,-0.31)	11	**738.1**	<0.001	CCC=0.11(-0.07,0.29)
LPS10	IFN	1	5	**14.8**	5	**572.0**	0.003		0			
		2	10	**727.9**	5	**1086.2**	0.701	rho=0.51(-0.03,0.82)	6	**3821.1**	0.06	rho=0.97(0.86,0.99)
		3	9	**257.2**	7	**3824.7**	0.005	CCC=0.02(-0.005,0.04)	6	**1957.1**	0.026	CCC=0.76(0.65,0.84)
	TNF	1	5	**166.3**	3	**9390.9**	<0.001		4	**3405.5**	0.001	
		2	10	**4026.3**	5	**4422.7**	0.903	INS	7	**1830.0**	0.24	rho=0.11(-0.60,0.72)
		3	9	**931.2**	5	**2831.5**	0.157		7	**2711.2**	0.124	CCC=0.05(-0.27,0.35)
	IL-10	1	16	**259.3**	10	**522.6**	0.143		10	**72.5**	0.002	
		2	20	**986.1**	16	**541.8**	0.124	rho=0.24(-0.10,0.53)	10	**83.4**	<0.001	rho=0.24(-0.12,0.55)
		3	16	**642.4**	10	**702.2**	0.852	CCC=0.23(-0.10,0.52)	12	**100.8**	<0.001	CCC=0.07(-0.04,0.18)
	IL-5	1	16	**1.8**	10	**3.2**	0.199		10	**1.8**	0.947	
		2	20	**2.0**	16	**3.3**	0.176	ND	10	**2.2**	0.808	ND
		3	16	**2.4**	10	**6.4**	0.026		12	**2.3**	0.872	
	IL-4	1	16	**2.7**	10	**3.3**	0.545		10	**2.3**	0.554	
		2	20	**2.4**	16	**1.6**	0.181	ND	10	**2.1**	0.654	ND
		3	16	**3.0**	10	**4.0**	0.406		12	**2.3**	0.299	
	IL-2	1	16	**4.9**	10	**8.5**	0.27		10	**3.4**	0.385	
		2	20	**3.7**	16	**7.4**	0.091	ND	10	**4.6**	0.578	ND
		3	16	**6.3**	10	**26.5**	0.004		12	**3.7**	0.203	
LPS100	IFN	1	5	**70.2**	5	**592.5**	0.078		0	.		
		2	12	**1283.7**	10	**4517.0**	0.126	rho=0.63(0.20,0.86)	6	**4803.8**	0.119	rho=0.99(0.94,1.00)
		3	9	**376.3**	6	**2821.1**	0.046	CCC=0.24(0.04,0.43)	6	**2105.5**	0.058	CCC=0.82(0.75,0.86)
	TNF	1	5	**2095.1**	1	**11504.7**	0.249		2	**5471.9**	0.386	
		2	10	**4644.1**	9	**3325.0**	0.615	INS	8	**3222.2**	0.575	rho=-0.32(-0.79,0.39)
		3	10	**1882.1**	5	**5430.8**	0.167		6	**3688.6**	0.334	CCC=-0.19(-0.53,0.20)
	IL-10	1	16	**610.2**	10	**718.0**	0.734		10	**123.2**	<0.001	
		2	19	**1121.9**	15	**388.4**	0.008	rho=0.29(-0.06,0.58)	15	**203.6**	<0.001	rho=0.11(-0.23,0.42)
		3	16	**802.6**	10	**1032.1**	0.599	CCC=0.14(-0.10,0.37)	12	**173.2**	<0.001	CCC=0.04(-0.08,0.15)
	IL-5	1	16	**2.5**	10	**3.9**	0.286		10	**1.8**	0.421	
		2	19	**2.0**	15	**3.6**	0.096	ND	15	**2.1**	0.764	ND
		3	16	**2.2**	10	**5.3**	0.047		12	**2.1**	0.875	
	IL-4	1	16	**3.4**	10	**2.8**	0.618		10	**2.3**	0.149	
		2	19	**2.5**	15	**2.9**	0.588	ND	15	**2.5**	0.97	ND
		3	16	**3.1**	10	**2.9**	0.842		12	**2.0**	0.082	
	IL-2	1	16	**7.7**	10	**8.6**	0.826		10	**3.3**	0.049	
		2	19	**3.9**	15	**9.7**	0.036	ND	15	**4.1**	0.909	ND
		3	16	**6.2**	10	**20.6**	0.016		12	**4.2**	0.334	
BLAG2	IFN	3	15	**79.7**	10	**1218.7**	<0.001		11	**67.8**	0.815	
		5	21	**229.8**	16	**211.1**	0.893	rho=-0.04(-0.37,0.30)	19	**179.7**	0.657	rho=-0.07(-0.36,0.24)
		7	14	**205.3**	10	**102.2**	0.378	CCC=-0.04(-0.36,0.29)	19	**112.5**	0.343	CCC=-0.06(-0.33,0.22)
	TNF	3	14	**389.3**	4	**2142.3**	0.027		10	**1685.3**	0.01	
		5	18	**218.4**	13	**339.7**	0.39	rho=0.54(0.15,0.79)	16	**1161.9**	<0.001	0.17(-0.17,0.48)
		7	13	**174.4**	8	**290.5**	0.419	CCC=0.54(0.16,0.78)	18	**547.9**	0.027	
	IL-10	3	16	**568.1**	10	**643.2**	0.795		12	**120.0**	<0.001	
		5	21	**219.5**	17	**58.2**	<0.001	rho=0.31(-0.02,0.57)	20	**82.7**	0.002	rho=-0.07(-0.35,0.22)
		7	15	**130.5**	11	**24.8**	<0.001	CCC=0.15(-0.01,0.30)	20	**41.3**	<0.001	CCC=0.13(-0.13,0.36)
	IL-5	3	16	**3.1**	10	**7.3**	0.05		12	**3.4**	0.731	
		5	21	**6.1**	17	**5.9**	0.933	ND	20	**6.0**	0.954	ND
		7	15	**7.3**	11	**7.4**	0.982		20	**10.4**	0.246	
	IL-4	3	16	**3.0**	10	**5.1**	0.138		12	**2.9**	0.841	
		5	21	**2.9**	17	**1.7**	0.053	ND	20	**2.6**	0.593	ND
		7	15	**2.7**	11	**1.6**	0.104		20	**3.4**	0.364	
	IL-2	3	16	**8.4**	10	**26.4**	0.022		12	**4.5**	0.13	
		5	21	**13.4**	17	**7.9**	0.191	rho=-0.17(-0.46,0.16)	20	**7.1**	0.066	ND
		7	15	**12.5**	11	**5.2**	0.074	CCC=-0.16(-0.44,0.15)	20	**11.2**	0.779	
DERF1	IFN	3	16	**9.8**	10	**28.9**	0.16		12	**4.5**	0.242	
		5	21	**16.8**	16	**18.3**	0.899	rho=0.40(0.08,0.64)	20	**8.8**	0.233	rho=0.43(0.16,0.65)
		7	13	**38.6**	10	**21.9**	0.48	CCC=0.40(0.09,0.63)	20	**12.7**	0.086	CCC=0.40(0.14,0.60)
	TNF	3	16	**193.2**	10	**204.4**	0.92		12	**79.5**	0.089	
		5	21	**27.0**	17	**67.4**	0.048	rho=0.39(0.08,0.63)	20	**58.6**	0.073	rho=0.18(-0.12,0.45)
		7	13	**23.0**	11	**139.1**	0.002	CCC=0.32(0.06,0.53)	20	**87.3**	0.009	CCC=0.16(-0.10,0.40)
	IL-10	3	16	**11.8**	8	**5.8**	0.169		12	**3.6**	0.002	
		5	21	**5.1**	17	**4.7**	0.83	rho=0.65(0.41,0.80)	20	**3.6**	0.289	ND
		7	13	**4.5**	11	**3.3**	0.526	CCC=0.60(0.37,0.76)	20	**4.4**	0.948	
	IL-5	3	16	**2.9**	10	**2.3**	0.592		12	**3.6**	0.531	
		5	21	**3.9**	17	**5.0**	0.465	ND	20	**3.8**	0.934	ND
		7	13	**8.3**	11	**8.4**	0.99		20	**2.7**	<0.001	
	IL-4	3	16	**2.7**	10	**3.3**	0.602		12	**2.6**	0.837	
		5	21	**2.8**	17	**1.7**	0.07	ND	20	**2.9**	0.857	ND
		7	13	**3.1**	11	**1.6**	0.053		20	**3.3**	0.824	
	IL-2	3	16	**20.8**	10	**23.0**	0.846		12	**7.5**	0.014	
		5	21	**32.8**	17	**10.4**	0.005	rho=0.06(-0.26,0.38)	20	**9.0**	<0.001	rho=0.32(0.03,0.56)
		7	13	**41.6**	11	**11.4**	0.011	CCC=0.05(-0.19,0.27)	20	**8.0**	<0.001	CCC=0.20(0.01,0.38)
