# Supplementary material for: Standardization and performance evaluation of mononuclear cell cytokine secretion assays in a multicenter study
Source: BMC Immunol. 2006 Dec 12;7:29. doi: 10.1186/1471-2172-7-29 (PMC1762025; doi:10.1186/1471-2172-7-29)
Supplement: Additional file 2 — Supplemental Table 2 – Cytokine from fresh and cryopreserved CBMC. Complete data set of secreted cytokine from fresh and cryopreserved CBMC culture. N = number of samples; (below limit) = number below detectable level for indicated stimulant/cytokine; SD = standard deviation; Rho = Pearson correlation; CCC = Concordance Correlation Coefficient. See methods for details of statistical analysis. [file 1471-2172-7-29-S2.pdf]

Supplemental Table 2. - Cytokine from fresh and cryopreserved CBMC.

Stimulant	cytokine	N (below limit)	Geometric mean-fresh (SD)	N (below limit)	Geometric mean-cryo (SD)	the ratio of Fresh/ CRF (95% confidence)	p-value	Rho	CCC
INNATE:									
MEDIUM	IFN-gamma	40(0)	7.96(1.31)	34(29)	7.6(1.39)	0.95(0.83,1.1)	0.498	-0.08(-0.41,0.26)	-0.06(-0.33,0.20)
	IFN-alpha	40(0)	8.86(1.28)	34(27)	7.48(1.32)	0.84 (0.75,0.95)	0.007	-0.06(-0.39,0.28)	-0.05(-0.33,0.23)
	TNF	40(38)	7.41(1.46)	34(29)	8.28(1.89)	1.12(0.88,1.42)	0.361	-0.08(-0.41,0.27)	-0.07(-0.36,0.24)
	IL-10	40(40)	6.8 (1)	34(32)	7.53 (1.52)	1.11 (0.97,1.26)	0.123		
	IL-12 p40	40(0)	12.82 (1.38)	34(0)	27.14 (2.01)	2.12 (1.66,2.71)	<0.001	0.07(-0.27,0.40)	0.02(-0.09,0.14)
	IL-12 p70	40(40)	6.8(1)	34(34)	6.8(1)				
	Rantes	40(0)	620.4 (2.11)	34(0)	1073.7 (1.71)	1.73 (1.27,2.35)	0.001	-0.11(-0.44,0.23)	-0.09(-0.35,0.18)
LPS	IFN-gamma	40(0)	8 (1.3)	38(32)	7.65 (1.39)	0.96 (0.84,1.09)	0.518	-0.01(-0.33,0.31)	-0.01(-0.32,0.29)
	IFN-alpha	40(0)	21.08 (2.78)	38(27)	7.65 (1.29)	0.36 (0.26,0.51)	<0.001	-0.24(-0.52,0.08)	-0.06(-0.13,0.02)
	TNF	40(0)	1279(2.02)	38(2)	105.5(3.43)	0.08(0.05,0.13)	<0.001	0.13(-0.20,0.43)	0.04(-0.06,0.14)
	IL-10	40(0)	137.27 (1.49)	38(7)	39.91 (3.24)	0.29 (0.2,0.43)	<0.001	0.47(0.17,0.68) **	0.10(0.03,0.17) **
	IL-12 p40	40(0)	103.36 (2.25)	38(0)	26.84 (2.11)	0.26 (0.18,0.37)	<0.001	-0.18(-0.47,0.15)	-0.07(-0.20,0.06)
	IL-12 p70	40(38)	6.87(1.05)	38(38)	6.8(1)				
	Rantes	40(0)	677.92 (1.5)	38(0)	991.72 (1.3)	1.46 (1.25,1.71)	<0.001	-0.52(-0.72,-0.24) ***	-0.28(-0.46,-0.08)
ADAPTIVE:									
MEDIUM	IFN-gamma	40(0)	7.52(1.12)	32(0)	13.14(1.19)	1.75(1.63,1.87)	<0.001	-0.13(-0.46,0.23)	-0.02(-0.06,0.03)
	IL-10	40(40)	6.8(1)	32(31)	6.93(1.11)	1.02(0.99,1.05)	0.267		
	IL-13	40(40)	6.8(1)	32(30)	7.47(1.45)	1.1(0.98,1.23)	0.112		
	IL-4	40(0)	8.12(1.02)	32(32)	6.8(1)	0.84(0.83,0.84)	<0.001		
	IL-5	40(40)	6.8(1)	32(32)	6.8(1)				
	VEGF	40(0)	48.96(2.57)	32(0)	19.04(1.61)	0.39(0.27,0.56)	<0.001	0.15(-0.21,0.47)	0.06(-0.08,0.19)
PHA	IFN-gamma	40(0)	707.79 (3.12)	38(0)	21.67(2.51)	0.03 (0.02,0.05)	<0.001	-0.49(-0.70,-0.21) **	-0.07(-0.12,-0.02)
	IL-10	40(0)	185.34 (3.26)	38(21)	8.96 (1.56)	0.05 (0.03,0.07)	<0.001	0.17(-0.15,0.47)	0.02(-0.02,0.05)
	IL-13	40(0)	240.65 (2.46)	38(11)	33.71 (3.95)	0.14 (0.08,0.24)	<0.001	0.58(0.32,0.76) ***	0.22(0.09,0.34) ***
	IL-4	40(40)	24.16 (2.45)	38(27)	7.92 (1.3)	0.33 (0.24,0.44)	<0.001	0.75(0.57,0.87) ***	0.17(0.09,0.25) ***
	IL-5	40(40)	6.8(1)	40(40)	6.8(1)				
	VEGF	40(0)	15.62 (1.35)	38(0)	15.6 (1.36)	1 (0.87,1.15)	0.984	-0.15(-0.45,0.18)	-0.15(-0.45,0.17)
